# Distinct Roles of Jasmonates and Aldehydes in Plant-Defense Responses

**DOI:** 10.1371/journal.pone.0001904

**Published:** 2008-04-02

**Authors:** E. Wassim Chehab, Roy Kaspi, Tatyana Savchenko, Heather Rowe, Florence Negre-Zakharov, Dan Kliebenstein, Katayoon Dehesh

**Affiliations:** 1 Section of Plant Biology, University of California Davis, Davis, California, United States of America; 2 Department of Plant Sciences, University of California Davis, Davis, California, United States of America; University of Melbourne, Australia

## Abstract

**Background:**

Many inducible plant-defense responses are activated by jasmonates (JAs), C_6_-aldehydes, and their corresponding derivatives, produced by the two main competing branches of the oxylipin pathway, the allene oxide synthase (AOS) and hydroperoxide lyase (HPL) branches, respectively. In addition to competition for substrates, these branch-pathway-derived metabolites have substantial overlap in regulation of gene expression. Past experiments to define the role of C_6_-aldehydes in plant defense responses were biased towards the exogenous application of the synthetic metabolites or the use of genetic manipulation of *HPL* expression levels in plant genotypes with intact ability to produce the competing AOS-derived metabolites. To uncouple the roles of the C_6_-aldehydes and jasmonates in mediating direct and indirect plant-defense responses, we generated *Arabidopsis* genotypes lacking either one or both of these metabolites. These genotypes were subsequently challenged with a phloem-feeding insect (aphids: *Myzus persicae*), an insect herbivore (leafminers*: Liriomyza trifolii*), and two different necrotrophic fungal pathogens (*Botrytis cinerea* and *Alternaria brassicicola*). We also characterized the volatiles emitted by these plants upon aphid infestation or mechanical wounding and identified hexenyl acetate as the predominant compound in these volatile blends. Subsequently, we examined the signaling role of this compound in attracting the parasitoid wasp (*Aphidius colemani*), a natural enemy of aphids.

**Principal Findings:**

This study conclusively establishes that jasmonates and C_6_-aldehydes play distinct roles in plant defense responses. The jasmonates are indispensable metabolites in mediating the activation of direct plant-defense responses, whereas the C_6_-aldehyes are not. On the other hand, hexenyl acetate, an acetylated C_6_-aldehyde, is the predominant wound-inducible volatile signal that mediates indirect defense responses by directing tritrophic (plant-herbivore-natural enemy) interactions.

**Significance:**

The data suggest that jasmonates and hexenyl acetate play distinct roles in mediating direct and indirect plant-defense responses. The potential advantage of this “division of labor” is to ensure the most effective defense strategy that minimizes incurred damages at a reduced metabolic cost.

## Introduction

Plants employ a complex array of physical and chemical defense mechanisms to resist or evade biotic attacks. In addition to the constitutive defense mechanisms such as trichomes, thick secondary wall or toxic compounds, plants are also equipped with inducible defense mechanisms [1], [2]. The inducible defenses function either directly via mechanisms such as production of amino acid catabolizing enzymes, antidigestive proteins, and toxic or repelling chemicals [3]–[5], or indirectly through production and release of volatile organic compounds (VOC) as a signal to the natural enemies of invaders that their prey is in the vicinity [6]–[10]. Many inducible defense responses are activated by oxylipins, the oxygenated derivatives of fatty acids generated via the oxylipin branch pathways [11], [12]. Allene oxide synthase (AOS) and hydroperoxide lyase (HPL) are the two main competing oxylipin-pathway branches producing stress-inducible compounds [13]. The metabolites of the AOS branch are jasmonates [jasmonic acid (JA), methyl jasmonate (MeJA) and their biosynthetic precursor, 12- oxophytodienoic acid (12-OPDA)]. The best characterized metabolites of the HPL branch are the green leafy volatiles (GLVs) that predominantly consist of C_6_-aldehydes [(*Z*)-3-hexenal, *n*-hexanal] and their respective derivatives such as (*Z*)-3-hexenol, (*Z*)-3-hexen-1-yl acetate, and the corresponding *E*-isomers [14].

The functional role of JAs in mediating plant defense responses has received far more attention than the HPL-derived metabolites [15]. To examine the defensive function of C_6_-aldehydes and their respective derivatives, investigators have altered the levels of GLVs either by the exogenous application of synthetic metabolites [16]–[20], or by genetic manipulation of the *HPL* expression levels in plant genotypes that are intact in their ability to produce the competing AOS-derived metabolites [8], [21]–[23]. Collectively, these studies provide strong support for the important role of the HPL-derived metabolites in mediating plant defense responses. However, given the well documented substrate competition between the two branch pathways [13], [14], and the considerable overlap in regulation of gene expression by HPL- and AOS-derived oxylipins [21], it has not been possible to conclusively determine whether or not each of these metabolites plays a distinct role in mediating direct and/or indirect plant defense responses. To uncouple the signaling roles of the C_6_-aldehydes from those of the jasmonates in defense responses, we have generated an ensemble of plant genotypes lacking either one or both metabolites, and subsequently challenged them with various invaders as well as an insect parasitoid. The outcome of these analyses clearly establishes that jasmonates mediate the direct plant-defense responses whereas, hexenyl acetate, an acetylated C_6_-aldehyde is the predominant wound-inducible volatile that mediates indirect defense responses by attracting the natural enemies of plant invaders to their prey.

## Results

### Hexenyl acetate is the predominant plant volatile synthesized *de novo* in a transient fashion in response to wounding

**Figure 1:**
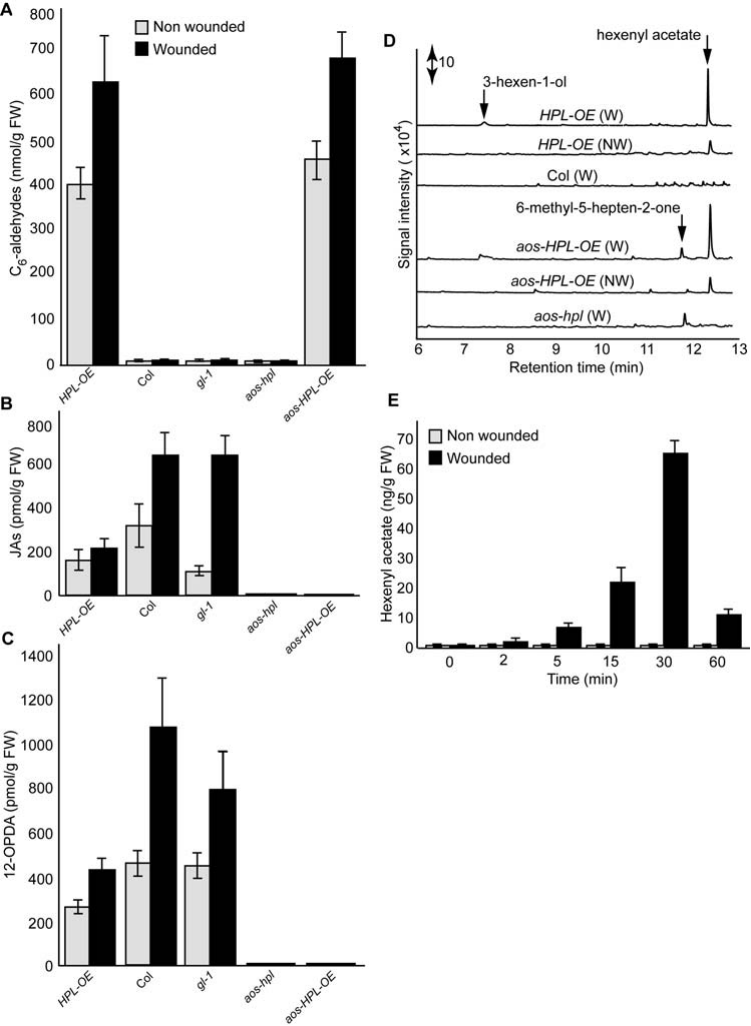
Profiling of the HPL- and AOS-branch pathways metabolites. (A) Levels of C_6_-aldehydes, (B) JAs (JA+MeJA), and (C) 12-OPDA determined in non wounded (grey bar), or wounded leaves 2 hours after mechanical damage (black bar). Each measurement is derived from the mean±standard deviation (SD) of three independent biological replicates. (D) Characterization and quantification of GLVs by adsorptive headspace collection and GC-MS analyses performed on three repeats of three independent biological replicates from wounded and non wounded *Arabidopsis* genotypes show that hexenyl acetate is the predominant volatile produced in wounded leaves of plants with a functional *HPL*. Double-headed arrow represents a scale for signal intensity. (E) Analyses of the emission rate of hexenyl acetate in non wounded (grey bar) or mechanically wounded (black bar) *aos-HPL-OE* plants, performed three times on three independent biological replicates show that emission of hexenyl acetate is wound-inducible and transient.

We generated an ensemble of plant genotypes lacking either one or both sets of AOS- and HPL-derived metabolites using natural genetic variation and transgenic technologies. The *Arabidopsis* accession Columbia-0, is a natural loss-of-function mutant in *hpl* and thereby lacks C_6_-aldehydes [24]. The double mutant lacking both C_6_-aldehydes and jasmonates (*aos-hpl*) is an engineered T-DNA insertion line in *AOS* resulting in generation of *aos* loss-of-function plants in the trichomeless background (*gl-1*, accession Col-0) [25]. Hence this plant genotype is impaired in its ability to accumulate both JAs and C_6_-aldehydes. In addition, this plant is male sterile and can only be maintained as homozygous for the *aos* mutation by spraying the developing flowers with MeJA [25]. We genetically modified these existing single and double mutant lines to produce C_6_-aldehydes. To restore the aldehyde-producing capabilities of the wild type Col (WT) background, we had previously generated transgenic plants overexpressing a rice *Os*HPL3-GFP fusion construct (*HPL-OE*), as well as lines expressing GFP alone as the control (for simplicity designated here as Col) [26]. The basal and wound induced levels of C_6_-aldehydes (hexenals and hexanals) in *HPL-OE* plants were at least 50-fold higher than the negligible levels produced via non enzymatic cleavage of the substrate in the control lines (Figure 1A). Wounding induces a 33% increase of these C_6_-aldehydes in the *HPL-OE* line (Figure 1A). This is in spite of the constitutive expression of *HPL* under the 35S promoter, which may indicate the limited availability of the basal levels of substrates. Concomitant with these increases in the levels of C_6_-aldehydes there is a ∼60% reduction in the basal and wound-induced levels of JAs (JA and MeJA) and 12-OPDA in the *HPL-OE* as compared to the levels in the control Col, the naturally *hpl* mutant background (Figure 1B & C). These data, consistent with the previous reports [21], confirm the controlling role of substrate flux in biosynthesis of oxylipins and demonstrate that overexpression of the HPL branch reduces the pool of substrate available for the biosynthesis of jasmonates.

To restore C_6_-aldehyde metabolism in the double mutant background (*aos-hpl*), while circumventing any potential influence of the transgene's insertion site or accumulation of second site mutations as the result of the transformation process, we out-crossed the *HPL-OE* to the *aos-hpl* and generated F_1_ lines. To select a homozygous *aos-HPL-OE* plant in the subsequent segregating populations, we exploited the male sterile phenotype observed in plants lacking jasmonates [25], in combination with the use of the selectable marker employed in generation of *HPL-OE* lines. Profiling of AOS- and HPL-derived metabolites of wounded and non wounded *aos-HPL-OE* homozygous plants determined that while their jasmonates are below detection levels, their C_6_-aldehydes are at levels comparable to those present in the *HPL-OE* line (Figure 1A, B, C). As a control we also out-crossed *aos-hpl* to Col, and generated a homozygous *aos-hpl*-*GFP* line for simplicity now designated also as *aos-hpl*. These plants, similar to the parental *aos* loss-of-function line in the *gl-1* background, are impaired in the production of both jasmonates and C_6_-aldehydes in contrast to the *gl-1* background that is deficient only in C_6_-aldehydes (Figure 1A, B, C).

To simultaneously characterize and quantify the wound induced VOCs, we conducted adsorptive headspace collection from all the above described genotypes. This analysis identified 3-hexen-1-yl acetate (hexenyl acetate), the acetylated derivative of (*Z*)-3-hexenol, as the predominant volatile which was released only from the aldehyde-producing plants namely, the *aos-HPL-OE* and *HPL-OE* lines (Figure 1D). Wounding of *aos-HPL-OE* or *HPL-OE* lines led to emission of ∼20-fold higher levels of hexenyl acetate than the corresponding non-wounded plants. Additional analyses designed to measure the emission rate of hexenyl acetate established that this plant volatile is synthesized *de novo* and is released rapidly and transiently in response to wounding. Specifically, these data show that a negligible basal level of hexenyl acetate is emitted from the non wounded plants (Figure 1E). However, 2 minutes after wounding these levels are increased by ∼2-fold (3 ng/g FW), reaching the maximum levels (68 ng/g FW) at 30 minutes, and declining by ∼6.5-fold (12 ng/g FW) by 60 minutes.

### Jasmonates and C_6_-aldehydes play distinct roles in mediating direct and indirect plant-defense responses

**Figure 2:**
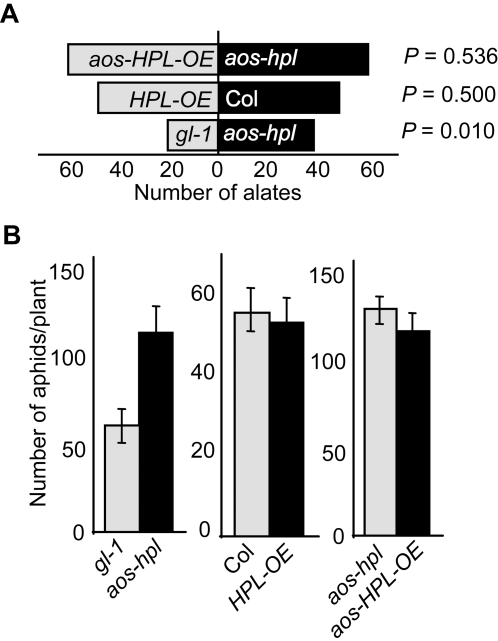
Choice and no choice tests with the green peach aphid (*Myzus persicae*). (A) Choice bioassays performed on pairs of plant genotypes where a single *M. persicae* alate female was released in each cage containing the most comparable pair of genotypes. The initial nymph deposition preference was determined within 2 days of aphid release. Bar graphs represent the actual numbers of alates. One-tailed binomial tests were used to determine significance (*P*<0.05). (B) Population increase of aphids (fecundity) upon the release of a newly deposited nymph on a single plant of indicated genotype during 15 days of reproduction. The graphs indicate the mean numbers of aphids per plant±SE. Each of the above-described tests was performed on ∼30 individual plants per genotype.

The above described collection of plant genotypes provided us with the tools necessary to uncouple the individual roles of the AOS- and HPL-derived metabolites in mediating defense responses. A series of choice and no choice bioassays were employed to challenge the plants with a phloem-feeding generalist herbivorous insect, the green peach aphid (*Myzus persicae*). Choice bioassays were performed on pairs of genotypes best suited for the comparative analyses. Thus pairs of *aos-HPL-OE* with *aos*-*hpl,* Col with *HPL-OE*, and *gl-1* with *aos-hpl* were caged together and each pair was challenged with alate (winged) female aphids (Figure 2A). These studies collectively established that only the AOS-derived metabolites, and not the HPL-derived ones, mediate plant-direct defense responses against this insect. Specifically, the data clearly demonstrate that this insect does not show a preference to *aos-HPL-OE* versus *aos*-*hpl* or to Col versus *HPL-OE* genotypes (Figures 2A). However, this insect significantly preferred *aos-hpl* over *gl-1* (*P = *0.01) (Figure 2A). We also performed no choice feeding tests and examined the weight gain and fecundity of aphids reared on these plant genotypes. These data were consistent with the results of the choice studies as we found no statistically significant differences in the weight gain of aphids that were reared on the Col versus *HPL-OE*, or reared on the *aos-hpl* (*gl-1* background) versus *aos-hpl* (Col background) plants (data not shown). In contrast, aphids reared on the *aos-hpl* plants gained 40% more weight than those on the *gl-1* plants (data not shown). In addition, the only statistically significant difference obtained from the aphid fecundity test was a ∼2-fold increase in the numbers of aphids per *aos-hpl* plants as compared to those on *gl-1* (Figure 2B). It is well established that plants deficient in the production or recognition of JAs are more susceptible to attacks by chewing insects [3], [27]–[29]. Our data, however, further expand the role of JAs in plant protection not only against chewing but also against phloem-feeding insects.

**Figure 3:**
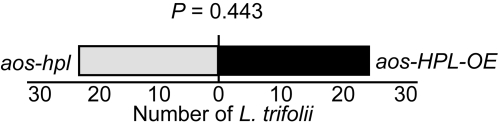
Choice test with the leafminer (*Liriomyza trifolii)*. Attraction of two-day old female leafminers to *aos-HPL-OE* versus *aos*-*hpl* plants was tested using glass Y-tube olfactometer. Each leafminer was introduced individually into the base of the Y-tube and its choice was recorded. The bar graph represents the numbers of herbivores examined and shows that they are equally attracted to the *aos*-*HPL-OE* and to the *aos-hpl* plants. One-tailed binomial tests were performed to determine the significance. (*P* = 0.443).

To determine whether other herbivore insects are attracted to the aldehyde producing plants, we also performed choice tests using a glass Y-tube olfactometer and compared attraction of leafminer (*Liriomyza trifolii)* females *to aos-hpl* versus *aos-HPL-OE* lines. These polyphagous females oviposit within the leaf, where the larvae feed and develop on the tissue [30]. This analysis showed that this herbivore is equally attracted to *aos-HPL-OE* and to *aso-hpl* lines (Figure 3).

**Figure 4:**
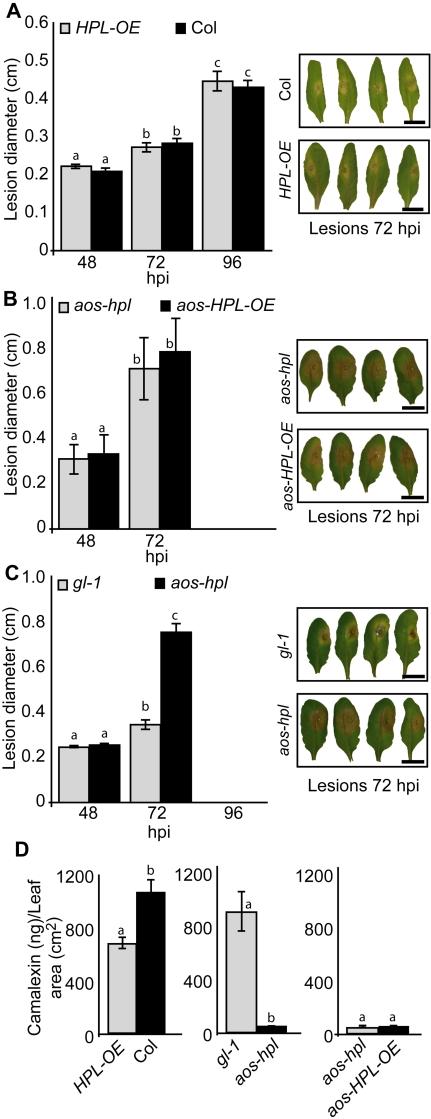
Function of JAs and C_6_-aldehydes in plant protection against necrotrophic fungus, *Botrytis cinerea.* Lesion development was monitored and compared between leaves isolated from (A) *HPL-OE* vs. Col, (B) *aos*-*hpl* vs. *aos-HPL-OE*, (C) *gl-1* vs. *aos-hpl*, at 48, 72 or 96 hours post inoculation (hpi) with *B. cinerea* conidia. Each bar graph represents average lesion diameter±SD of 24 inoculated leaves. All leaves lacking jasmonates (*aos-hpl*, *aos-HPL-OE)* show larger lesions as compared to those with a functional AOS (Col, *HPL-OE*, *gl-1*). The lesion sizes were not affected by the presence or absence of HPL-derived metabolites. For comparison, representative photographs of each genotype 72 hpi is shown. Graphs are the means±SD of 24 replicates for each genotype. Bar = 1 cm. (D) Analyses of camalexin accumulation levels for leaves collected at 72 hpi (*gl-1*, *aos-hpl* and *aos-HPL-OE*) or 96 hpi (Col and *HPL-OE*) show negligible levels of camalexin in all plant genotypes with dysfunctional *AOS.* The *HPL-OE* lines contain 30% less camalexin than that in Col lines, potentially because of the reduced JA levels in these plants. Graphs are the means±SD of 24 replicates for each genotype. Within any given treatment, bars with different letters indicate significant differences (*P*<0.005, Tukey's test).

To examine the function of JAs and C_6_-aldehydes in plant protection against necrotrophic pathogens we used conidia from the grey mold causing fungus, *Botrytis cinerea*, and inoculated the leaves of the above described collection of *Arabidopsis*. Measurements of the mean diameter of the necrotic area at different hours post inoculation (hpi) show that infection caused similar lesion size on Col versus *HPL-OE*, as well as on *aos-hpl* versus *aos-HPL-OE* (Figure 4A & B). The only notable difference is a ∼2-fold larger necrotic lesion on leaves of *aos-hpl* than that of *gl-1* (Figure 4C). Leaves from plants lacking JAs were completely infected at 96 hpi, thus measurements at this time point were not possible. To identify the underlying mechanisms for the observed compromised resistance in the *aos-hpl* mutant, we also examined the levels of camalexin, the main phytoalexin in *Arabidopsis* shown to inhibit growth of some *Botrytis cinerea* isolates [31]. The requirement of the JA-dependent signaling pathway for camalexin biosynthesis is well documented [32], [33], but such a potential role for the HPL-dependent signaling pathway is not yet reported. Hence, we examined the camalexin levels in *HPL-OE* and Col at 96 hpi, and determined that these levels in the *hpl-*loss of function Col mutant are comparable to those reported for other *Arabidopsis* accessions [31]. However, the *HPL-OE* lines contain significantly lower levels of camalexin (∼30%) than that in Col lines (Figure 4D). This reduction is potentially attributable to the ∼60% decrease in the JAs levels in *HPL-OE* as compared to Col lines (Figure 1B &C). Quantification of camalexin in *gl-1* versus *aos-hpl*, and in *aos-hpl* versus *aos-HPL-OE*, at 72 hpi show that the levels are drastically reduced in all plants lacking JAs irrespective of presence or absence of HPL-derived metabolites.

**Figure 5:**
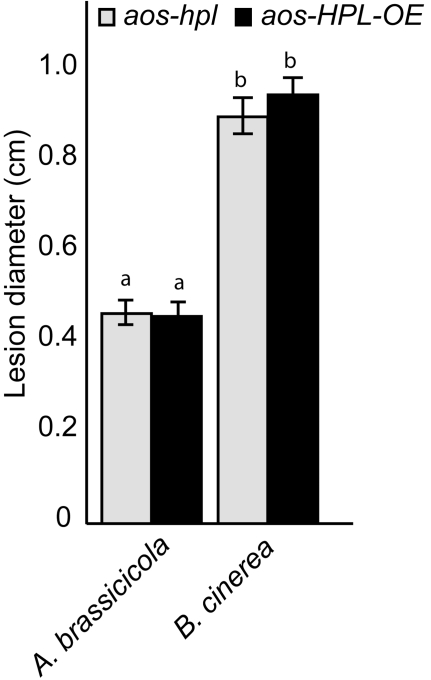
Function of C_6_-aldehydes in plant protection against necrotrophic fungi *Botrytis cinerea and Alternaria brassicicola*. Lesion development was monitored and compared between leaves from *aos-hpl* vs. *aos-HPL-OE* plants at 72h post inoculation (hpi) with conidia from either *B. cinerea* or from *A. brassicicola.* Each bar represents average lesion diameter±SD of 24 replicates for each genotype.

To determine whether this observed lack of enhanced resistance of *HPL-OE* line to necrotrophic fungi is limited to *B. cinerea,* we extended our analysis and used conidia from the black mold causing fungus, *Alternaria brassicicola,* and inoculated the leaves of *aos-hpl* and *aos-HPL-OE* plants. To exclude any potential experimental and/or environmental variations that might influence the data, we performed parallel inoculation of detached leaves from the same plant by conidia from *B. cinerea*. Subsequently, after 72 hpi, we measured mean diameter of lesions and determined that necrotic lesions caused by *B*. *cinerea* are ∼2-fold larger than those resulted from *A. brassicicola* (Figure 5). However in spite of this difference in lesion size, consistent with the previous data (Figure 4A–C) there were no detectable differences between susceptibility of *aos-HPL-OE* and *aos-hpl* plants to both these fungi (Figure 5).

### Hexenyl acetate is the volatile signal from plants to natural enemies of aphids

**Figure 6:**
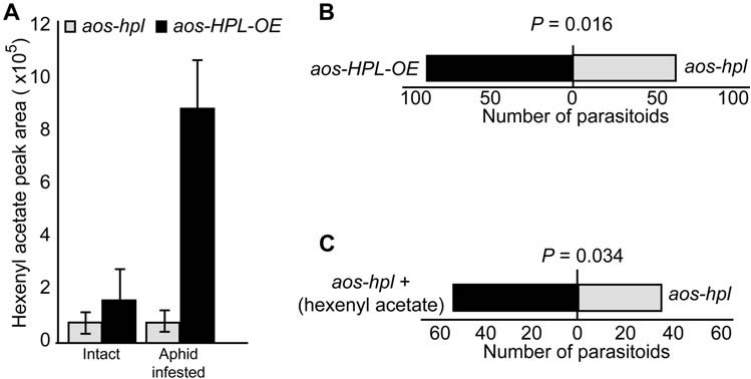
Attraction of parasitoid wasp, *Aphidius colemani,* to the *in vivo* wound-induced or chemically synthesized hexenyl acetate. (A) Characterization and quantification of GLVs by adsorptive headspace collection and GC-MS analyses performed on three repeats of five independent biological replicates from intact and aphid infested *aos-hpl* and *aos-HPL-OE* genotypes show that hexenyl acetate is the predominant volatile produced in aphid infested plants with a functional *HPL*. (B) Volatile bioassays using glass Y-tube olfactometer was employed to determine the response of *A. colemani* to the volatile blend produced from mechanically wounded *aos-hpl* and *aos-HPL-OE* plant genotypes. The bar graph represents the number of parasitoids examined and shows that they are significantly attracted more to the wounded *aos*-*HPL-OE* than to the *aos-hpl* plants (*P* = 0.016). (C) Volatile bioassays using glass Y-tube olfactometer was employed to determine the response of *A. colemani* to the presence or absence of synthetic hexenyl acetate in chambers containing wounded *aos-hpl* plant genotype. The bar graph represents the number of parasitoids examined and shows that they are significantly attracted towards the chamber of wounded *aos-hpl* plants with hexenyl acetate-spotted filters as compared to the plant chamber containing the same plant genotype but with hexane-spotted filters as the control (*P* = 0.034). One-tailed binomial tests were used to determine significance.

To characterize VOCs produced by aphid infested plants we conducted adsorptive headspace collection from intact and infested *aos-hpl* and *aos-HPL-OE* plants. Similar to the data obtained from mechanically wounded leaves (Figure 1D), these analyses also identified hexenyl acetate as the prevalent volatile that is predominantly released from the *aos-HPL-OE* plants infested with aphids (Figure 6A). To further examine the role of aldehydes in general and hexenyl acetate in particular in mediating plant indirect responses, we performed volatile bioassays using a glass Y-tube olfactometer and examined attraction of *Aphidius colemani*, to wounded *aos-hpl* versus *aos-HPL-OE*. We chose *Aphidius colemani* because this wasp is parasitic to a range of aphids including green peach aphid. The female wasp finds aphid colonies from a long distance by “alarm signals” produced by an infected plant and lays its egg directly inside the aphid, where the larva feeds and develops into a fully formed wasp killing the aphid in the process. Mechanically wounded plants were used for these experiments because wounding initiates an instantaneous and synchronous response that results in the generation of similar VOC profiles as those produced by aphid infestation. Preference tests using 160 *A. colemani* females released individually show that a statistically significant number of these female parasitoid wasps are attracted to *aos-HPL-OE*, as compared to *aos-hpl* plants (*P* = 0.016) (Figure 6B). To specifically examine the role of hexenyl acetate, the predominant wound-induced volatile among the complex blend emitted by the plants, we also performed volatile bioassays with wounded *aos-hpl* in the presence or absence of chemically synthesized hexenyl acetate. As a control, we also performed these tests with chemically synthesized MeJA. The wasps showed no preference to chemically synthesized MeJA (data not shown), but 60% of them were attracted to the jar containing *aos-hpl* plants along with the filters spotted with synthetic hexenyl acetate (*P* = 0.034) (Figure 6C).

## Discussion

Plants are constantly challenged with a wide spectrum of biotic stimuli to which they respond directly and/or indirectly through activation of complex signaling cascades among them the oxylipin pathway. The role of oxylipin pathway metabolites, mainly jasmonates and C6 aldehydes, the AOS- and HPL-derived metabolites respectively, in plant defense response has been documented [8], [16]–[23]. However to date the distinct roles played by each of these pathway metabolites in mediating direct- and indirect-defense reponses has remained elusive. To address this deficiency, we have generated an ensemble of plant genotypes lacking either one or both metabolites, and determined that even transgenic plants overexpressing *HPL* under a constitutive promoter exhibit an induction in the corresponding levels of metabolites upon wounding. As expected, this finding further confirms the controlling role of substrate flux in biosynthesis of oxylipins.

Further examination of the function of JAs and C_6_-aldehydes in plant protection against invaders led to unexpected results. Specifically, in contrast to previous reports [21], [22], HPL-derived metabolites played no role in either the attraction or development of the aphids, nor did they attract or repel the herbivore, leafminer. The role of C_6_-aldehydes in mediating direct defense responses was previously demonstrated by a 2-fold increase in fecundity of aphids reared on potatoes with reduced HPL activity as compared to those feeding on wild type plants [22]. Furthermore, a different response to C_6_-aldehydes was observed with three lepidopteron herbivores of *Nicotiana attenuata* where these compounds function as feeding stimulants for the larvae [21]. The discrepancy between our finding and these previous reports could be explained by different scenarios influencing the co-evolutionary traits central to the plant-insect interaction, such as species-specific compounds, ratios of ubiquitous metabolites, or different species-species responses [34]. In contrast to the ineffectiveness of HPL-derived metabolites in resistance to aphids, plants deficient in the production of JAs are more susceptible to attacks by the same insect herbivore. This finding further expands direct role of JAs in plant protection against both chewing insects [3], [27]–29, as well as sucking insect.

Evaluation of the role of these oxylipins in providing plant protection against two necrotrophic pathogens, *B. cineara* and *A. brassicicola*, further illustrate the indispensable function of jasmonates, and not C_6_-aldehyes in mediating the activation of direct plant-defense responses. As expected, overexpression of HPL branch of the oxylipin cascade reduced the pool of available substrates for JAs biosynthesis which, in turn led to reduced levels of camalexin whose biosynthesis require the JA-dependent signaling pathway. Interestingly however, the reduced camalexin levels in HPL-OE lines did not enhance their susceptibility to these pathogen infections, indicating that these levels are above the threshold necessary to potentiate resistance. Previous reports indicated that exposure of *Arabidopsis* to synthetic (*E*)-2-hexenal and (*Z*)-3-hexenal increased camalexin levels and simultaneously enhanced plant resistance against *B. cinerea* [17]. To date there is no direct evidence to conclusively demonstrate that the exogenous application of C_6_-aldehydes induces the same responses as those of *in vivo* produced metabolites. Therefore the basis of this mismatch between the two data sets may be due to differential responses of plants to exogenous application versus the *in vivo* generated C_6_-aldehydes. More surprisingly, our data does not match the findings conducted on *Arabidopsis* plants with modulated levels of aldehydes [23]. These investigators found that lines expressing an *HPL* anti-sense construct displayed a 70% decrease in C_6_-aldehydes and a 10% increase in lesion size with *Botrytis cinerea*. Considering the DNA homology between *Arabidopsis AOS* and *HPL*, it is conceivable that the use of a full-length anti-sense HPL cDNA clone used by these investigators to silence *HPL* expression may have led to the down regulation of both the *HPL* and *AOS* transcripts, however neither JA levels nor AOS expression levels were measured in these experiments. Under such a scenario the larger lesions in these plants may be due to decreased levels of JAs rather than reduced levels of aldehydes. Another possibility is that there is a difference between the two *Botrytis cinerea* genotypes used for their sensitivity to C_6_-aldehydes or C_6_-aldehyde regulated defenses. In the same study two lines constitutively overexpressing a Bell Pepper HPL produced 12% or 25% more C_6_-aldehydes than the wild type upon *Botrytis* infection, while the respective basal levels of these metabolites remained unchanged as compared to that of the wild type. *Botrytis* lesions in these two independent transgenics were identical in size and were slightly (∼7–8%) smaller than that of wild type. Although the basis of the mismatch between these data and our findings is unclear, both studies suggest a lack of linear correlation between lesion size and alteration of C_6_-aldehyde levels. Given this and the difference in pathogen resistance in JA deficient lines, led us therefore to conclude that C_6_-aldehydes at the very least are not major contributors to direct defenses against either *B. cinerea* or *A. brassicicola.*

While the VOC profiles show no detectable levels of the AOS-derived metabolite, both mechanically wounded and aphid infested HPL-expressing plants emit substantial levels of hexenyl acetate, the predominant volatile released in response to these abiotic and biotic stimuli. This similarity in VOC profiles in wounded and aphid infested plants is a manifestation of a similar metabolic landscape that is the outcome of the large overlap reported to exist between abiotic and biotic stress-responsive genes [35]. Additional analyses designed to measure the emission rate of hexenyl acetate established that this plant volatile is synthesized *de novo* and is released rapidly and transiently in response to wounding. This suggests that the biosynthesis of hexenyl acetate is delicately balanced and tightly regulated as a potential strategy to effectively reduce the metabolic cost in response to wounding. This finding further corroborates the role of induction in protecting plants from the adverse impact of high metabolic cost on further growth and development supporting the cost-benefit model of induced resistance [36]. In addition, this transient release of volatiles versus a constitutive one would provide specific and directional signaling to attract predators of the plant invaders. The absence of detectable levels of MeJA in the VOC blend suggests that this compound is not a general defense response volatile in *Arabidopsis*.

Examination of the role of hexenyl acetate in directing tritrophic (plant-herbivore-natural enemy) interactions unequivocally demonstrates that this volatile is the chemical cue emitted from the plants to attract the parasitoid wasp. Our observations are consistent with recent reports on the importance of GLVs in recruitment of the herbivores natural enemies [23], [37]. Recent identification of an insect neuron responding to 6 GLVs, including hexenyl acetate, through employment of gas chromatography linked to electrophysiological recording from single receptor neurons (RN) [38], further strengthens the direct role of hexenyl acetate in attracting the parasitoid wasp.

In summary this communication establishes the distinct biological roles played by the AOS- and HPL-competing branch pathway-metabolites and highlights the importance of this “division-of-labor” strategy in providing effective protection against invaders at reduced metabolic costs. In addition, the plant genotypes generated in this work will provide valuable genetic tools for further dissection of the intricate and complex interplay of oxylipin-mediated signaling networks regulating the direct and/or indirect plant defense responses.

## Materials and Methods

### Plant lines and growth conditions

All transgenic and mutant *Arabidopsis thaliana* plants employed in this report were in Columbia-0 background (Col-0) and grown as previously described [26]. All the described experiments were performed with 5 week-old plants unless otherwise noted. The *gl-1* seeds [39] were kindly provided by Dr. Tom Jack (Dartmouth College, Hanover, NH). Seeds for the *aos* plants (CS6149), which were generated in *gl-1* background as a result of a T-DNA insertion in the *AtAOS* (*aos-ko)*, were purchased from the *Arabidopsis* Biological Resource Center (Columbus, OH). We performed PCR analyses and further confirmed the presence of T-DNA insertion within *AtAOS* as previously described [25]. In order to generate *Col-GFP* plants, here designated as Col, the open reading frame of GFP from pEZS-NLGFP vector was cut with *Not* I restriction enzyme and the generated DNA fragment was subsequently subcloned into the binary vector pMLBart, kindly provided by Dr. John Bowman (Monash University, Australia). Upon verification of the DNA insert by sequencing, the construct was used to transform Col-0. In addition, the *Os*HPL3-GFP construct in pMLBart previously described in [26] was used to transform Col-0 and to generate *HPL-OE* plants. Transformation was performed by the floral-dip method [40], and the *Agrobacterium* strain used was EHA101. T_1_ plants were germinated on soil. Selection of transgenics was by treating 10- to 12-d-old seedlings with 1∶1,000 Finale (the commercial product that is 5.78% glufosinate ammonium) twice a week. Surviving plants were further screened to select for transgenics containing single inserts which were further propagated to get the homozygous lines used in this report.

To obtain *aos-hpl-GFP*, also designated as *aos-hpl*, and *aos-HPL-OE* plants, pollen from homozygous Col and *HPL-OE* were used to fertilize the male sterile *aos-ko* flowers. Homozygous lines of *aos-hpl* and *aos-HPL-OE* were generated from the segregating F_1_ population using kanamycin as well as glufosinate ammonium as selection markers. All transgenes were verified by a number of approaches including PCR analyses using gene-specific primers as described below, in concert with the examination of male sterile phenotype and metabolic profiling of jasmonates and aldehydes, the products of the AOS and HPL branches respectively. All *Arabidopsis* lines containing a T-DNA insertion within the *AtAOS* were confirmed by PCR as previously described [25]. The following primers were further used to verify the presence of the *HPL-OE* transgene (5′-ATGGTGCCGTCGTTCCCGCA-3′ and 5′-TTAGCTGGGAGTGAGCTC-3′) and the *GFP* transgene (5′-ATGGTGAGCAAGGGCGAGGA-3′ and 5′-TACTTGTACAGCTCGTCCATGCCGAGAGT-3′).

Upon flowering, plants containing the *aos* genotype were sprayed every other day with 2 mM MeJA (Sigma) dissolved in 0.03% Silwett in order to maintain homozygous *aos-ko* and permit an otherwise male sterile plant to produce seeds.

### Sources of insects and their maintenance

Green peach aphid (*M. persicae*) colonies were maintained on cabbage seedlings (*Brassica oleracea* var. *capitata*) at laboratory conditions (25±5°C, 50±20% relative humidity, 16 h light). Leafminer (*L. trifolii*) flies were taken from a laboratory colony established on faba bean (*Vicia faba* L.), by collecting flies from infested gerbera plants from southern California. *A. colemani* pupae were obtained from Koppert Inc. (Netherlands). The parasitoids emerged in closed containers at the above described laboratory conditions employed for the development of aphids.

### Quantification of AOS- and HPL-derived metabolites

Extraction of JAs (MeJA and JA) as well as 12-OPDA were carried out as previously described [41], [42] with minor adjustments. In brief, leaf material (∼300 mg fresh weight) was collected from intact plants, quickly weighed, and immediately frozen in liquid nitrogen to minimize wound-induced accumulation of oxylipins. Samples were finely ground in mortar while frozen and transferred to a 4 ml screw top Supelco vial containing 1200 µl of 2-propanol/H_2_O/HCl (2∶1∶0.002) and sonicated in a water bath for 10 min. Dichloromethane (2 ml) was added to each sample and re-sonicated for 10 min. The bottom dichloromethane/2-propanol layer was then transferred to a 4 ml glass vial, evaporated under a constant air stream and the resultant pellet was subsequently dissolved in 300 µl of diethyl ether/methanol (9:1, vol/vol) followed by the addition of 9 µl of a 2.0 M solution of trimethylsilyldiazomethane in hexane in order to convert the carboxylic acids into the methyl esters. During this step JA is converted to MeJA. The vials were then capped, vortexed, and incubated at room temperature for 25 min. Then 9 µl of 12% acetic acid in hexane were added to each sample and left at room temperature for another 25 min in order to destroy all excess trimethylsilyldiazomethane. The above-mentioned procedure was also used to derivatize carefully calculated amounts of JA (Sigma Inc.) as well as 12-OPDA (Larodan Fine Chemicals Inc., Sweden) in triplicates to generate calibration curves used for the quantification of jasmonates.

The produced methyl ester volatiles were captured on Super-Q (Alltech Inc., State College, PA) columns by vapor-phase extraction as described [42]. The trapped metabolites were then eluted with 150 µl of dichloromethane and analyzed by GC-MS using a Hewlett and Packard 6890 series gas chromatograph coupled to an Agilent Technologies 5973 network mass selective detector operated in electronic ionization (EI) mode. One µl of the sample was injected in splitless mode at 250°C and separated using an HP-5MS column (30 m×0.25 mm, 0.25 µm film thickness) held at 40°C for 1 min after injection, and then at increasing temperatures programmed to ramp at 15°C/min to 250°C (10 min), with helium as the carrier gas (constant flow rate 0.7 ml/min). Measurements were carried out in selected ion monitoring (SIM) mode with retention times and M^+^ *m/z* ions as follows: JA-ME (*trans* 12.66 min, *cis* 12.91 min, 224) and 12-OPDA-ME (*trans* 18.31 min, *cis* 18.75 min, 306).

C_6_-aldehydes were measured exactly as previously described [26].

### Aphid dual-choice assays

Dual-choice assays were performed to identify the plant(s) on which aphids prefer to deposit their nymphs. Female alates were transferred using a fine hair brush and released into the center of a soil-containing pot, with an arena of 55×25 mm, in which 15 day-old test plants from the two most comparable genotypes (*aos-HPL-OE* with *aos*-*hpl,* Col with *HPL-OE*, and *gl-1* with *aos-hpl)* were grown on each half of the pot. After aphid transfer, they were kept at laboratory conditions and examined every 24 h for 3 successive days. The location of the first deposited nymphs was recorded. One-tailed binomial tests were performed to test the significance of the aphids' choices for nymph deposition [43].

### Aphid development assays

One newly deposited aphid nymph was transferred with a fine hair brush to a 2-week-old plant of a specific genotype, as described in this report. These young plants were used for this assay to give ample time for the newly-hatched nymph in each arena to develop and reproduce over a period of 15 days after which the plants on which they are fed were over 4-weeks old. Individual plants were confined in a thrips-screen cage and kept at laboratory conditions. Aphids developed for 15 days, and then they were collected and frozen at −20°C. The fecundity was determined by recording the total number of aphids offspring present on each plant. Subsequently adult aphids were put in an oven at 60°C for three days. Their dry weight was individually determined. *t*-tests were performed to compare the dry weights of the aphids, and Mann–Whitney rank sum tests when the assumptions for parametric tests were violated [43].

### Fungal pathogenicity tests and analysis of camalexin content

*B. cinerea* isolate ‘Grape’ was obtained from the laboratory of Melanie Vivier (University of Capetown, South Africa) [44]. *Alternaria brassicicola* isolate ‘AbBOB1’ was isolated from cauliflower at the University of California Cooperative Extension facility in Salinas, CA. Preparation of inoculum and infection of *Arabidopsis* plants was as previously described [45]. Mature rosette leaves excised from 5-week old *Arabidopsis* plants were placed in 145×20 mm plastic Petri dishes filled with 1% phytagar. Each dish contained a single genotype. Each experiment used at least 3 dishes per genotype, containing 8 leaves per dish. Leaves were inoculated with 4 µl droplets of 2.5×10^4^ conidia/ml in half-strength filtered organic grape juice (Santa Cruz Organics, CA) and incubated at room temperature. Lesion area (cm^2^) was measured from digital images (118 pixels/cm) of infected leaves using Image J [46] with scale objects included in images. Camalexin was extracted from individual infected leaves and quantified as described [31].

### Adsorptive headspace collection and analyses of volatiles emitted from wounded or aphid infested plants

GLVs collections were performed on ∼2.2 g of either non wounded or mechanically wounded 5 week-old *Arabidopsis* plants in ∼4 L glass desiccators-style containers (Duran Inc., Germany**)**. GLVs were also collected from plants that were either intact or infested with ∼500 aphid/plant. These plants were maintained, for the duration of sample collection (72 h), in ∼4 L glass desiccators-style containers.

The dynamic headspace collection was performed using an air pump, circulating charcoal purified air in a closed loop at a rate of ∼2 L min^−1^. Emitted volatiles were trapped in a filter containing 50 mg of Porapak Q® (Waters Inc., Milford, MA) at the indicated times and the metabolites were subsequently eluted by applying 200 µl of dichloromethane to the filter. GLVs were analyzed on the same GC-MS instrument described above. One µl of the eluted sample was injected at 250°C in splitless mode and separated on a DB1MS (30 m×0.25 mm×0.25 µm). The GC oven temperature was programmed as follows: 5 min at 40°C, ramp to 200°C at 6°C/min with no hold time, but with a post run of 5 min at 250°C. Helium was the carrier gas at 53 ml/min. The mass spectrometer was run in the scan mode. Triplicate measurements from three independent biological samples were carried out for each time point. The identity of (*Z*)-3-hexen-1-yl acetate was determined by comparing the retention time (12.68 min) and mass spectra with that of an authentic standard. The amount of the volatile was computed subsequent to careful preparation of a calibration curve using (*Z*)-3-hexen-1-yl acetate as a standard.

### Y-tube olfactometer bioassay using leafminer and parasitioid wasp

The following bioassay was performed as previously described [47] but with minor adjustments. Briefly, a glass Y-tube (diameter: 2.5 cm; trunk: 26 cm; arm: 12 cm) was used as the bioassay arena. The gas carrying the volatiles was clean in-house air which was filtered through activated charcoal before being split in two. Each stream was passed at 400 ml/min through ∼4 L glass container having 2.2 g of mechanically wounded *Arabidopsis* leaves. All connections between the parts described were with Teflon tubing. After every fourth run, the Y-tube and glass vessels washed and rinsed with acetone and placed in an oven at 60°C. All bioassays were carried out at room temperature under artificial lighting in a white cardboard box with the Y-tube vertically placed.

In order to test the response of the leafminer (*Liriomyza trifolii*) to HPL-derived volatiles we tested insect attraction to *aos-*HPL-OE versus *aos*-*hpl* plants. In these tests, one pot with 5 plants was placed within each glass container. One to two day-old female *L. trifolii*, assumed to have mated, were collected and used for this bioassay. Each leafminer was introduced individually into the base of the Y-tube and its choice was recorded. Each leafminer female was used only once. One-tailed binomial tests were performed to test the significance of the predators' choices for nymph deposition [43].

In order to test the response of *A. colemani* to HPL-derived metabolites, volatiles from wounded *aos*-*hpl* leaves were tested against those of *aos-HPL-OE*. In addition, the parasitoid's response to synthetic hexenyl acetate was tested by allowing it to choose between volatiles from wounded *aos*-*hpl* leaves placed next to filters spotted with either 100 ng of synthetically pure hexenyl acetate (10 ng/µl in hexane) or 10 µl of hexane as the control. One-tailed binomial tests were performed to test the significance of the predators' choices for nymph deposition [43].

Insects used for this bioassay were one day-old female *A. colemani*, assumed to have mated. Each parasitoid was introduced individually into the base of the Y-tube and its choice was recorded if it crossed 9.5 cm beyond the junction region. To control for directional bias the arms of the Y-tube through which the odor sources were presented, as well as the sources' locations, were swapped after every four parasitoids tested. Each parasitoid was used only once.
